# Intelligent Reflecting Surface Assisted Secure Transmission in UAV-MIMO Communication Systems

**DOI:** 10.3390/e24111605

**Published:** 2022-11-04

**Authors:** Tianhao Cheng, Buhong Wang, Zhen Wang, Kunrui Cao, Runze Dong, Jiang Weng

**Affiliations:** 1 School of Information and Navigation, Air Force Engineering University, Xi’an 710077, China; 2 School of Information Engineer, Xijing University, Xi’an 710123, China; 3 School of Information and Communications, National University of Defense Technology, Wuhan 430035, China; 4 State Key Laboratory of Integrated Services Networks, Xidian University, Xi’an 710071, China

**Keywords:** intelligent reflecting surface (IRS), unmanned aerial vehicle (UAV), alternating optimization (AO), secure communication

## Abstract

This paper studies the intelligent reflecting surface (IRS) assisted secure transmission in unmanned aerial vehicle (UAV) communication systems, where the UAV base station, the legitimate receiver, and the malicious eavesdropper in the system are all equipped with multiple antennas. By deploying an IRS on the facade of a building, the UAV base station can be assisted to realize the secure transmission in this multiple-input multiple-output (MIMO) system. In order to maximize the secrecy rate (SR), the transmit precoding (TPC) matrix, artificial noise (AN) matrix, IRS phase shift matrix, and UAV position are jointly optimized subject to the constraints of transmit power limit, unit modulus of IRS phase shift, and maximum moving distance of UAV. Since the problem is non-convex, an alternating optimization (AO) algorithm is proposed to solve it. Specifically, the TPC matrix and AN covariance matrix are derived by the Lagrange dual method. The alternating direction method of multipliers (ADMM), majorization-minimization (MM), and Riemannian manifold gradient (RCG) algorithms are presented, respectively, to solve the IRS phase shift matrix, and then the performance of the three algorithms is compared. Based on the proportional integral (PI) control theory, a secrecy rate gradient (SRG) algorithm is proposed to iteratively search for the UAV position by following the direction of the secrecy rate gradient. The theoretic analysis and simulation results show that our proposed AO algorithm has a good convergence performance and can increase the SR by 40.5% compared with the method without IRS assistance.

## 1. Introduction

Due to their low cost, high mobility, and easy deployment, unmanned aerial vehicles (UAVs) have been widely used in logistics transportation, earthquake relief, aerial search and rescue, etc. In addition, UAVs can act as air base stations, relays or user nodes, and play important roles in wireless communication. Compared with traditional terrestrial communication, on the one hand, UAVs can use more sophisticated three-dimensional (3D) beamforming technology to greatly improve channel capacity; on the other hand, UAVs have a high probability of forming line-of-sight (LoS) links with ground users with good channel quality. However, these characteristics of UAVs can also pose security challenges to wireless networks, as it is also easier for eavesdroppers to form LoS links with transmitters and perform passive eavesdropping or active attacks. Therefore, the security problem in UAV communication systems is a research content worthy of attention. However, traditional encryption techniques require high computational complexity and consume large amounts of energy, which are not suitable for energy-constrained UAV platforms.

As a powerful supplement to the upper layer encryption techniques, the physical layer security (PLS) technology uses the physical layer characteristics of the channel itself to improve the secrecy performance. While ensuring the secure and reliable communication of the legitimate receiver, it tries to avoid the effective eavesdropping of eavesdroppers [1,2,3,4]. PLS technology in UAV communication networks can achieve secure transmission with low energy consumption through the differentiated design of wireless channels. It does not require key management and distribution, and omits the encoding and decoding process, making it suitable for resource-constrained UAV communication platforms. Therefore, it has a high application prospect and research value.

The PLS of UAV communication networks has been widely investigated. Mamaghani and Hong considered the power allocation optimization problem between the confidential signal and the artificial noise (AN) signal transmitted by the UAV base station [5]. Ji et al. regarded the cache-enabled UAV as a reliable relay and proposed an optimization problem of maximizing the minimum SR among users [6]. For the case where the eavesdropper location obeys the Poisson point process, Sun et al. derived a closed expression for the lower bound on the average secrecy rate (ASR) and maximized it [7]. When only the statistical illegitimate channel state information (CSI) was known, Bao et al. derived a closed expression for the secrecy outage probability (SOP) and the ergodic SR [8], and Yuan et al. optimized the UAV trajectory and beamforming vector [9]. Dong et al. considered the coordinate multiple points technology to form a UAV swarm relay, by jointly optimizing the transmit power of the base station and UAV relay, power allocation coefficient and beamforming on UAV relays, and the trajectory to maximize the ASR [10]. Ye et al. considered that the UAV base station serves the legitimate UAV users under the eavesdropping of illegal UAVs, and derived the closed expressions of SOP and average secrecy capacity [11]. Wang et al. investigated the cooperation of high-altitude platform and UAVs to provide services for ground users, and jointly optimized channel allocation, users’ power, and UAVs’ three-dimensional (3D) position in the NOMA-enabled network to counter an eavesdropping UAV [12].

### 1.1. Related Work

Although the above PLS technologies have been studied in-depth, when the legal channel quality is further deteriorated or the energy consumption of the communication node is limited, the above techniques may not meet the needs of secure communication. Therefore, the emerging intelligent reflecting surface (IRS) technology is introduced into the design of the secure UAV communication system. By integrating a large number of low-cost passive reflection components and controlling each amplitude and phase to reflect the incident signal independently, IRS can achieve a passive 3D beamforming, which can modify the wireless propagation environment and bring a higher degree of design freedom to secure wireless communications [13,14]. Wang et al. deployed an IRS on the UAV as a trusted relay to maximize the ASR by jointly optimizing the beamforming vector, IRS phase shift matrix, and UAV’s trajectory [15]. Sun et al. optimized the positions and beamforming of a UAV base station and IRS deployed on building walls to maximize the SR [16], and Pang et al. further optimized the trajectory of UAVs [17]. In addition, Fang et al. optimized the transmission power of the base station [18]. In contrast, Li et al. considered a time division multiple access communication system [19], and Li et al. extended this problem to a multi-user scenario [20]. In addition to the convex optimization methods used in the above research, Guo et al. used the deep deterministic policy gradient (DDPG) framework and proposed a twin-DDPG deep reinforcement learning algorithm to solve the SR optimization problem [21].

Although the above works have carried out a certain degree of research on IRS-assisted UAV secure communication, they are all based on the multiple-input single-output (MISO) channel or single-input single-output (SISO) channel and do not involve the problems of UAV-assisted multiple-input multiple-output (MIMO) scenarios. In a MIMO system, multiple parallel data streams can be transmitted at the same time to increase channel capacity, and the spatial multiplexing gain and spatial diversity gain can be used to overcome the channel fading, which has obvious advantages compared with MISO and SISO systems. However, due to the difference of channel models, the optimization problems in MIMO system are much more complicated than that in MISO systems. Firstly, the beamforming vector optimization in MISO systems needs to be transformed into covariance matrix optimization in MIMO systems. Secondly, the expression of the achievable rate is in the form of the logarithm of scalars in MISO communication systems, while in MIMO communication systems, the achievable rate expression takes the form of the logarithm of matrices determinant. This means that the optimization objectives, constraints, and optimization techniques are different from those in MISO systems, which is more challenging to deal with. Especially in the optimization of deployment position of the UAV, the strong non-convexity of the optimization problem makes it difficult to apply similar algorithms such as the successive convex approximation (SCA) and the semi-definite programming (SDP) to MIMO scenarios, and therefore difficult to solve using traditional convex optimization methods.

Therefore, current works have conducted in-depth research on the PLS communication of UAV-MISO systems, but only a few of them involve MIMO scenarios: considering the impact of multi-antenna eavesdroppers on UAV communication, Maeng et al. proposed a new linear precoder design scheme for data and AN transmission and derived a closed expression for the ASR for cellular connected UAVs networks [22], but they did not address the optimal design of deployment position of the UAV. Yuan et al. studied the secure beamforming and UAV trajectory planning problems in MIMO transceiver and multi-antenna eavesdropper (MIMOME) scenarios [9], but this research used the exhaustive search method of discrete processing, which has a large amount of calculation and low accuracy and thus makes it easy to lose the optimal solution. Mamaghani et al. proposed a full-duplex UAV relay scheme based on AN to maximize the ASR, but the reinforcement learning method requires high hardware cost and is not suitable for environmental changes [23]. However, the above research does not involve the booming IRS technology and does not fully exploit the secure communication capabilities of UAV-MIMO systems.

### 1.2. Main Contributions

As mentioned above, the current various research works mainly focus on the secure transmission of UAV-MISO systems. The related research on the PLS of UAV-MIMO communication is still in its infancy currently. In particular, there is still a research gap in the effect of IRS on the PLS of UAV-MIMO systems, and the corresponding design of the optimization algorithm is not reported in the literature yet, which motivates this work. In this paper, we jointly optimize the UAV position, transmit precoding (TPC) matrix and AN matrix, and IRS phase shift matrix to maximize the SR of an IRS assisted UAV-MIMOME communication system. The formulated problem is non-convex, and it is quite difficult to convert it into a convex problem for an approximate solution using common methods such as the successive convex approximation (SCA) or fractional programming. Therefore, we propose an alternating optimization (AO) algorithm to deal with this problem and obtain the suboptimal solution through multiple iterations. The main contributions of this paper are summarized as follows.

(1) Different from the above literatures which focused MISO communication [15,16,17,18,19,20,21], we utilize the IRS to enhance the security of the UAV-MIMOME wireless communication system. Specifically, the system is composed of a UAV base station, an IRS, a ground legal receiver, and a ground eavesdropper. Each node is equipped with multiple antennas. The SR of the UAV communication system is maximized by jointly optimizing the TPC matrix and AN matrix, the IRS phase shift matrix, and the UAV placement subjected to transmit power constraint, unit modulus constraint, and maximum moving distance constraint within each iteration interval.
(2) Since the optimization problem is non-convex, an AO algorithm is designed to solve it. Specifically, using the weighted minimum mean square error (WMMSE) algorithm to convert the original problem into a tractable equivalent form. For the optimization of the TPC matrix and AN matrix, we introduce auxiliary matrices and solve their expressions by the Lagrange dual method. For the optimization of the IRS phase shift matrix, after the problem is transformed into a constrained quadratically constrained quadratic program (QCQP) problem, three methods of alternating direction method of multipliers (ADMM), majorization-minimization (MM), and Riemannian manifold gradient (RCG) are used to solve it. For the optimization of the UAV placement, existing research based on MISO channels [24,25] and traditional convex optimization methods for other parameters [26,27,28]–such as UAV coverage or outage probability–cannot be directly applied to MIMO scenarios with more complex channel models. Therefore, we propose a secrecy rate gradient (SRG) method, which combines the change of the SR with the UAV’s proportional integral (PI) control theory, so that the UAV moves towards the position with greater SR until it reaches the point with the maximum SR.
(3) The simulation results verify the advantages of the proposed algorithm compared with the benchmark schemes. It can be seen that the proposed AO algorithm can guide the UAV to move closer to the IRS, which proves that the IRS can effectively improve the security of the UAV communication system. In addition, increasing the transmission power and the number of antennas at legitimate nodes are beneficial to improve the secrecy performance. Moreover, the channel fading coefficients also play an important role in secure UAV-MIMO communications.

The rest parts of this paper are organized as follows. In Section 2, the system model and the optimization problem are formulated. In Section 3, we propose an AO algorithm to solve the optimization problem, which alternately solves the three sub-problems of the UAV position, TPC matrix, and AN matrix, and IRS phase shift matrix. In Section 4, we present the simulation results to verify the effectiveness of the proposed algorithms. We conclude this paper in Section 5.

Notations: In this paper, matrices and column vectors are denoted by bold uppercase letters and bold lowercase letters, Tr(⋅) and det(⋅) represent trace and determinant. ${\left(\cdot \right)}^{T}$, ${\left(\cdot \right)}^{H}$, ${\left(\cdot \right)}^{*}$, and ${\left(\cdot \right)}^{†}$ denote the transpose, Hermitian, conjugate, and pseudo-inverse operators, respectively. ${\mathbb{C}}^{M\times 1}$ denotes the space of M-dimensional complex-valued column vector.

## 2. System Model and Problem Formulation

### 2.1. System Model

**Figure 1:**
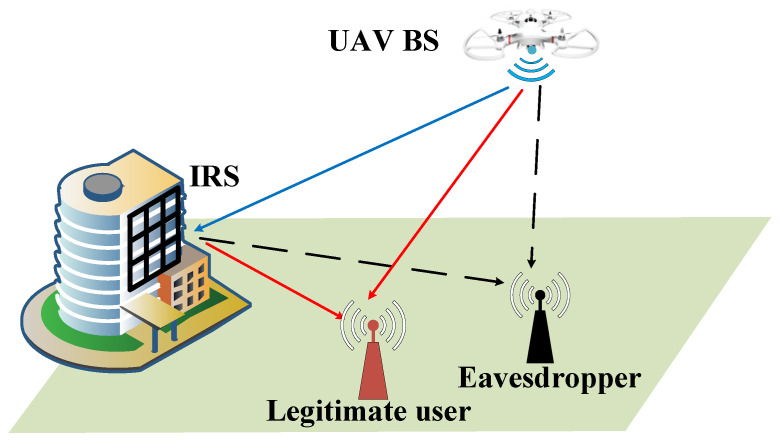
An IRS-assisted secure ground-to-air communication system.

As shown in Figure 1, we consider a MIMOME air-to-ground communication system, where the UAV base station equipped with a linear array transmits the information to the ground legitimate user Bob. Simultaneously, a ground eavesdropper Eve tries to eavesdrop on confidential information. To get close to Bob and stay away from Eve, the UAV can actively move to find the best location for secure communication. All nodes are equipped with multiple antennas. The number of transmit antennas at the UAV is $N_{T}$, and the numbers of receive antennas at the Bob and Eve are $N_{B}$ and $N_{E}$, respectively. In order to enhance the secrecy capacity, an IRS is deployed on the facade of a building to assist the communication between the UAV and Bob, which reflects the transmit signal of the UAV to increase the legitimate channel gain and damage the wiretap channel. The IRS consists of $M=M_{X}\times M_{Y}$ reflecting elements. Define the IRS phase shift matrix as $\Theta =\mathrm{diag}\left\{\theta_{1},\ldots ,\theta_{m},\ldots ,\theta_{M}\right\},\forall m\in M$, where $\theta_{m}=e^{j\phi_{m}},\phi_{m}\in [0,2\pi ]$. Assuming that the element spacing of each antenna array is half wavelength, namely, $d=\lambda_{c}/2$.

Without loss of generality, we consider a 3D Cartesian coordinate communication system. The UAV’s location is denoted as $p_{A}={\left[x_{A},y_{A},z_{A}\right]}^{T}$, and the coordinates of Bob, IRS, and Eve are $p_{B}={\left[x_{B},y_{B},z_{B}\right]}^{T}$, $p_{R}={\left[x_{R},y_{R},z_{R}\right]}^{T}$, and $p_{E}={\left[x_{E},y_{E},z_{E}\right]}^{T}$, respectively.

The channel gains between the UAV and ground receivers consist of LoS and non-LoS (NLoS) components. Therefore, we assume that the air-to-ground channel adopts the Rician fading channel model, and its channel gain depends on the Rician factor. In this paper, we assume that the transmitter can obtain the perfect CSI of all communication nodes, including the eavesdropper’s CSI. This assumption is possible, for example, when eavesdroppers are also legitimate users of the network, but they are not supposed to receive certain information, and they should be considered passive eavesdroppers. In this case, these undesired users can feed back perfect CSI to the transmitter. Based on existing IRS-aided communication channel estimation methods [29], we can directly focus on the final optimization task [15,16,17,18]. Therefore, this paper assumes that all CSI is available. The channel gain between the UAV and terrestrial nodes, denoted by $H_{Ai}^{}$, is given by

(1) $$H_{Ai}^{T}=\sqrt{\beta_{0}d_{Ai}^{-c_{Ai}}}\cdot {\tilde{H}}_{Ai}^{T}=\sqrt{\beta_{0}d_{Ai}^{-c_{Ai}}}(\sqrt{\frac{k_{Ai}}{k_{Ai}+1}}H_{Ai}^{LoS}+\sqrt{\frac{1}{k_{Ai}+1}}H_{Ai}^{NLoS}),i\in B,E,$$

where $\beta_{0}$ is the reference channel gain at distance 1 m, $d_{ab}^{}$ denotes the distance, $k_{ab}$ is the Rician factor between node a and b, $c_{ab}$ is the path loss exponent for a to b link. $H_{Ai}^{\mathrm{LoS}}$ represents the deterministic LoS component, and $H_{Ai}^{\mathrm{NLoS}}$ represents the random scattering component. Let $\phi_{i}$ and $\varphi_{i}$ denote the azimuth angle-of-arrival (AOA) and angle-of-departure (AOD), and let $\vartheta_{i}$ and $\theta_{i}$ denote the elevation AOA and AOD, respectively. The adjacent antenna distance on the transmitter and IRS array is d, then $H_{Ai}^{\mathrm{LoS}}$ can be expressed as

(2) $$H_{Ai}^{\mathrm{LoS}}=h_{Ai}^{\mathrm{LoS}(A)}\cdot h_{Ai}^{\mathrm{LoS}(D)},$$

where

(3) $$h_{Ai}^{\mathrm{LoS}(A)}={\left[1,e^{-j\frac{2\pi }{\lambda_{c}}d\sin\vartheta_{Ai}\cos\phi_{Ai}},\ldots ,e^{-j\frac{2\pi }{\lambda_{c}}(N_{i}-1)d\sin\vartheta_{Ai}\cos\phi_{Ai}}\right]}^{T},$$

(4) $$h_{Ai}^{\mathrm{LoS}(D)}=\left[1,e^{-j\frac{2\pi }{\lambda_{c}}d\sin\theta_{Ai}\cos\varphi_{Ai}},\ldots ,e^{-j\frac{2\pi }{\lambda_{c}}(N_{T}-1)d\sin\theta_{Ai}\cos\varphi_{Ai}}\right].$$

The random scattering component $H_{Ai}^{\mathrm{NLoS}}$ is the NLoS component, which is modeled by the circularly symmetric complex Gaussian (CSCG) distribution with zero mean and unit variance.

Similarly, we assume that the channel between the UAV and IRS contains LoS and NLoS components, so the Rician fading channel model is adopted, which can be expressed as

(5) $$H_{AR}=\sqrt{\beta_{0}d_{AR}^{-c_{AR}}}\cdot {\tilde{H}}_{AR}=\sqrt{\beta_{0}d_{AR}^{-c_{AR}}}h_{AR}^{\mathrm{LoS}(A)}h_{AR}^{\mathrm{LoS}(D)},$$

where $h_{AR}^{\mathrm{LoS}(A)}$ and $h_{AR}^{\mathrm{LoS}(D)}$ are given by

(6) $$h_{AR}^{\mathrm{LoS}(A)}={\left[1,e^{-j\frac{2\pi }{\lambda_{c}}d\sin\vartheta_{AR}\cos\phi_{AR}},\ldots ,e^{-j\frac{2\pi }{\lambda_{c}}d\sin\vartheta_{AR}((M_{X}-1)\cos\phi_{AR})},\ldots ,e^{-j\frac{2\pi }{\lambda_{c}}d\sin\vartheta_{AR}((M_{X}-1)\cos\phi_{AR}+(M_{Y}-1)\sin\phi_{AR})}\right]}^{T},$$

(7) $$h_{AR}^{\mathrm{LoS}(D)}=\left[1,e^{-j\frac{2\pi }{\lambda_{c}}d\sin\theta_{AR}\cos\varphi_{AR}},\ldots ,e^{-j\frac{2\pi }{\lambda_{c}}(N_{T}-1)d\sin\theta_{AR}\cos\varphi_{AR}}\right].$$

In addition, the channel gains between IRS and terrestrial nodes can be expressed as

(8) $$H_{Ri}^{T}=\sqrt{\beta_{0}d_{Ri}^{-c_{Ri}}}\cdot {\tilde{H}}_{Ri}^{T}=\sqrt{\beta_{0}d_{Ri}^{-c_{Ri}}}\cdot (\sqrt{\frac{k_{Ri}}{k_{Ri}+1}}h_{Ri}^{LoS(A)}\cdot h_{Ri}^{LoS(D)}+\sqrt{\frac{1}{k_{Ri}+1}}H_{Ri}^{NLoS}),i\in \left\{B,E\right\},$$

where $H_{Ri}^{\mathrm{NLoS}}$ follows CSCG distribution, and

(9) $$h_{Ri}^{\mathrm{LoS}(A)}={\left[1,e^{-j\frac{2\pi }{\lambda_{c}}d\sin\theta_{Ri}\cos\varphi_{Ri}},\ldots ,e^{-j\frac{2\pi }{\lambda_{c}}(N_{i}-1)d\sin\theta_{Ri}\cos\varphi_{Ri}}\right]}^{T},$$

(10) $$h_{Ri}^{\mathrm{LoS}(D)}=\left[1,e^{-j\frac{2\pi }{\lambda_{c}}d\sin\theta_{Ri}\cos\varphi_{Ri}},\ldots ,e^{-j\frac{2\pi }{\lambda_{c}}d\sin\theta_{Ri}((M_{X}-1)\cos\varphi_{Ri})},\ldots ,e^{-j\frac{2\pi }{\lambda_{c}}d\sin\theta_{Ri}((M_{X}-1)\cos\varphi_{Ri}+(M_{Y}-1)\sin\varphi_{Ri})}\right].$$

In order to achieve the secure transmission of confidential information, with the help of multiple antennas, the UAV focuses the confidential signal to Bob by generating a TPC matrix, while sending AN signal to Eve to damage its eavesdropping quality. The transmitted signal can be written as

(11) x=Vs+z,

where $V\in {\mathbb{C}}^{N_{T}\times N_{d}}$ represents the precoding matrix, $N_{d}\leq \min\left(N_{T},N_{B}\right)$ denotes the number of data streams, $s∼CN(0,I_{N_{d}})$ represents the transmitted signal, and z∼CN(0,Z) denotes the AN vector with zero mean and covariance matrix Z. Therefore, the received signals of the legitimate user and eavesdropper can be expressed as

(12) $$y_{i}=\left(H_{Ri}^{H}\Theta H_{AR}+H_{Ai}^{H}\right)x+n_{i},i\in \{B,E\},$$

where $n_{i}∼CN(0,\sigma_{i}^{2}I_{N_{i}}),i\in \{B,E\}$.

### 2.2. Quadcopter UAV Model

Define $c^{\left(n\right)}$ and $u^{\left(n\right)}$ as the state vector and input vector of the quadrotor UAV control system at a given iteration time n, respectively, and A and B respectively represent the influence of the state vector and the input vector on the derivative of each element in the state vector. The control system of quadcopter UAV is modeled as the following matrix differential equation,

(13) $$\Sigma :\left\{\begin{array}{l} {\dot{c}}^{\left(n\right)}=Ac^{\left(n\right)}+Bu^{\left(n\right)}\\ {\dot{c}}^{\left(0\right)}=c_{0} \end{array}\right.,$$

We take the square of the speed of the four rotors of the quadrotor UAV as the input vector $u^{\left(n\right)}$, and the state vector contains four parts: the UAV position, the orientation, and the derivative of the two–namely, $c=(p_{A}^{T},o_{A}^{T},{\dot{p}}_{A}^{T},{\dot{o}}_{A}^{T})$. The direction vector is expressed as $o_{A}={\left(-\theta_{A},\phi_{A},\psi_{A}\right)}^{T}$, where $\theta_{A}$, $\phi_{A}$ and $\psi_{A}$ represent pitch angle, roll angle, and yaw angle, respectively. We adopt the linear model in [30] to express this UAV system as

(14) $$\left\{\begin{array}{c} \begin{array}{ll} \dot{s}(n & )=\left(\begin{array}{ccccc} 0_{6\times 3} & 0_{6\times 1} & 0_{6\times 1} & 0_{6\times 1} & I_{6\times 6}\\ 0_{1\times 3} & g & 0 & 0 & 0_{1\times 6}\\ 0_{1\times 3} & 0 & g & 0 & 0_{1\times 6}\\ 0_{4\times 3} & 0_{4\times 1} & 0_{4\times 1} & 0_{4\times 1} & 0_{4\times 6} \end{array}\right)s(n)\\ & +(\begin{array}{c} 0_{8\times 4}\\ ϒ \end{array})u(n)+(\begin{array}{c} 0_{11\times 1}\\ g \end{array}) \end{array}\\ \;s(0)=s_{0} \end{array}\right.,$$

where g is the gravity constant and ϒ is a specific full-rank matrix. We only consider the horizontal position change of UAV. When the input signal is selected as $u(n)={ϒ}^{-1}{\left(u_{1}^{\left(n\right)}\;u_{2}^{\left(n\right)}\;u_{z}^{\left(n\right)}\;0\right)}^{T}$, the system model in the horizontal direction can be expressed as

(15) $$\Sigma_{\gamma }:\left\{\begin{array}{l} {\dot{c}}_{A,\gamma }^{\left(n\right)}=(\begin{array}{cccc} 0 & 1 & 0 & 0\\ 0 & 0 & g & 0\\ 0 & 0 & 0 & 1\\ 0 & 0 & 0 & 0 \end{array})c_{A,\gamma }^{\left(n\right)}+(\begin{array}{c} 0\\ 0\\ 0\\ 1 \end{array})u_{\gamma }^{\left(n\right)}\\ c_{A,\gamma }^{\left(0\right)}=c_{A,\gamma ,0} \end{array}\right.,$$

where $\gamma \in \left\{1,2\right\}$ represent the X-axis direction and the Y-axis direction, and

(16) $$c_{A,\gamma }^{\left(n\right)}=(\begin{array}{c} p_{A,\gamma }^{\left(n\right)}\\ {\dot{p}}_{A,\gamma }^{\left(n\right)}\\ o_{A,\gamma }^{\left(n\right)}\\ {\dot{o}}_{A,\gamma }^{\left(n\right)} \end{array}).$$

### 2.3. Problem Formulation

Based on the above analysis and to facilitate the solution, we further define the AN matrix $Z=V_{E}V_{E}^{H}$, $V_{E}\in {\mathbb{C}}^{N_{T}\times N_{T}}$, and set $H_{B}=\sigma_{B}^{-1}\left(H_{RB}^{H}\Theta H_{AR}+H_{AB}^{H}\right)$, and $H_{E}=\sigma_{E}^{-1}\left(H_{RE}^{H}\Theta H_{AR}+H_{AE}^{H}\right)$, the achievable rates of the legitimate user and eavesdropper can be expressed, respectively, as

(17) $$R_{B}=\log_{2}\left|I_{N_{B}}+H_{B}VV^{H}H_{B}^{H}J_{B}^{-1}\right|,$$

(18) $$R_{E}=\log_{2}\left|I_{N_{E}}+H_{E}VV^{H}H_{E}^{H}J_{E}^{-1}\right|,$$

where $J_{B}^{}=H_{B}V_{E}V_{E}^{H}H_{B}^{H}+I_{N_{B}}$, and $J_{E}^{}=H_{E}V_{E}V_{E}^{H}H_{E}^{H}+I_{N_{E}}$. Then, the SR $R_{\sec}$ is the difference between the two achievable rates. We aim to maximize the SR by jointly optimizing the precoding matrix V, AN matrix Z, IRS phase shift matrix Θ, and UAV position $p_{A}$. Therefore, the SR maximization problem of the UAV communication system can be formulated as

(19) $$\begin{array}{ll} \underset{V,V_{E},\Theta ,p_{A}}{\max} & R_{\sec}={\left[R_{B}-R_{E}\right]}^{+}\\ \;s.t. & \mathrm{Tr}\left(VV^{H}+V_{E}V_{E}^{H}\right)\leq P_{\max}\\ & \left|\theta_{m}\right|=1,\forall m\in M\\ & \left\|p_{A}(n)-p_{A}(n-1)\right\|\leq \delta ,\forall n\in N, \end{array}$$

where $P_{\max}$ is the maximum transmit power. The first constraint is the transmit power limit, the second constraint is the phase shift matrix unit modulus constraint, and the last constraint is the UAV moving distance limit between iterations.

## 3. Proposed Solution for Joint Optimization

In this section, we propose an AO algorithm to solve the Equation (19). The problem is decomposed into three subproblems; the precoding matrix V and AN matrix Z, the IRS phase shift matrix Θ, and the UAV position $p_{A}$ are solved alternately. Since this optimization problem is non-convex and thus difficult to solve, we need to transform it into an easy-to-solve form. Following the work of Hong et al. on IRS aided secure MIMO communication [31], we convert the objective function of Equation (19) into the following form:

(20) $$\begin{array}{l} R_{\sec}=\log_{2}\left|I_{N_{B}}+H_{B}VV^{H}H_{B}^{H}J_{B}^{-1}\right|-\log_{2}\left|I_{N_{E}}+H_{E}VV^{H}H_{E}^{H}J_{E}^{-1}\right|\\ \;=\underset{f_{1}}{\underset{︸}{\log_{2}\left|I_{N_{B}}+H_{B}VV^{H}H_{B}^{H}{\left(I_{N_{B}}+H_{B}V_{E}V_{E}^{H}H_{B}^{H}\right)}^{-1}\right|}}\\ \;\underset{f_{2}}{\underset{︸}{+\log\left|I_{N_{E}}+H_{E}V_{E}V_{E}^{H}H_{E}^{H}\right|}}\underset{f_{3}}{\underset{︸}{-\log\left|I_{N_{E}}+H_{E}\left(VV^{H}+V_{E}V_{E}^{H}\right)H_{E}^{H}\right|}}{\;}_{\cdot } \end{array}$$

The problem is still an intractable non-convex problem that requires further reformulation. As for $f_{1}$, we transmit the SR maximation problem to another equivalent problem by introducing the weighted minimum mean square error (WMMSE) method [32]. By introducing the linear decoding matrix $Z_{1}\in {\mathbb{C}}^{N_{T}\times d}$, the MSE matrix of $f_{1}$ is

(21) $$\begin{array}{l} \;E_{1}(Z_{1},V,V_{E})≜E_{s,z,n_{B}}\left[(\hat{s}-s){\left(\hat{s}-s\right)}^{H}\right]\\ =\left(I_{d}-Z_{1}^{H}H_{B}V\right){\left(I_{d}-Z_{1}^{H}H_{B}V\right)}^{H}+Z_{1}^{H}\left(I_{N_{B}}+H_{B}V_{E}V_{E}^{H}H_{B}^{H}\right)Z_{1}. \end{array}$$

Introducing the slack variable $X_{1}\in {\mathbb{C}}^{d\times d}$, and using Lemma 4.1 in [33], $f_{1}$ can be reformulated as

(22) $$f_{1}=\underset{Z_{1},X_{1}≽0}{\max}h_{1}\left(Z_{1},V,V_{E},X_{1}\right)=\underset{Z_{1},X_{1}≽0}{\max}\log\left|X_{1}\right|-\mathrm{Tr}\left(X_{1}E_{1}(Z_{1},V,V_{E})\right)+N_{d}.$$

Then the optimal $Z_{1}$ and $X_{1}$ can be expressed as:

(23) $$\begin{array}{ll} Z_{1}^{*} & =\arg\underset{Z_{1}}{\max}h_{1}\left(Z_{1},V,V_{E},X_{1}\right)=\arg\underset{Z_{B}}{\min}\mathrm{Tr}\left(X_{1}E_{1}(Z_{1},V,V_{E})\right)\\ & ={\left(I_{N_{B}}+H_{B}V_{E}V_{E}^{H}H_{B}^{H}+H_{B}VV^{H}H_{B}^{H}\right)}^{-1}H_{B}V, \end{array}$$

(24) $$\begin{array}{ll} X_{1}^{*} & =\arg\underset{X_{1}≽0}{\max}h_{1}\left(Z_{1},V,V_{E},X_{1}\right)={\left[E_{1}(Z_{1}^{*},V,V_{E})\right]}^{-1}\\ & =\left[\left(I_{N_{d}}-Z_{1}^{*}{}^{H}H_{B}V\right)\left(I_{N_{d}}-Z_{1}^{*}{}^{H}H_{B}V\right)\right.{\left.+Z_{1}^{*}{}^{H}\left(I_{N_{B}}+H_{B}V_{E}V_{E}^{H}H_{B}^{H}\right)Z_{1}^{*}\right]}^{-1}. \end{array}$$

Similarly, by introducing the linear decoding matrix $Z_{2}\in {\mathbb{C}}^{N_{E}\times N_{T}}$ and the slack variable $X_{2}\in {\mathbb{C}}^{N_{T}\times N_{T}}$, we can get the MSE matrix of $f_{2}$, and $f_{2}$ can be reformulated as

(25) $$E_{2}(Z_{2},V_{E})≜\left(I_{N_{T}}-Z_{2}^{H}H_{E}V_{E}\right){\left(I_{N_{T}}-Z_{2}^{H}H_{E}V_{E}\right)}^{H}+Z_{2}^{H}Z_{2},$$

(26) $$f_{2}=\underset{Z_{2},X_{2}≽0}{\max}h_{2}\left(Z_{2},V_{E},X_{2}\right)=\underset{Z_{2},X_{2}≽0}{\max}\log\left|X_{2}\right|-\mathrm{Tr}\left(X_{2}E_{2}(E_{2},V_{E})\right)+N_{T}.$$

Then the optimal $Z_{2}$ and $X_{2}$ can be expressed as

(27) $$Z_{2}^{*}=\arg\underset{Z_{2}}{\max}h_{2}\left(Z_{2},V_{E},X_{2}\right)={\left(I_{N_{E}}+H_{E}V_{E}V_{E}^{H}H_{E}^{H}\right)}^{-1}H_{E}V_{E},$$

(28) $$\begin{array}{ll} X_{2}^{*} & =\arg\underset{X_{2}≽0}{\max}h_{2}\left(Z_{2},V_{E},X_{2}\right)={\left[E_{2}(Z_{2}^{*},V_{E})\right]}^{-1}\\ & ={\left[\left(I_{N_{T}}-Z_{2}^{*}{}^{H}H_{E}V_{E}\right){\left(I_{N_{T}}-Z_{2}^{*}{}^{H}H_{E}V_{E}\right)}^{H}+Z_{2}^{*}{}^{H}Z_{2}^{*}\right]}^{-1}. \end{array}$$

As for $f_{3}$, we need to introduce Lemma 1 in [34] for reformulation,

(29) $$f_{3}=\underset{X_{3}≽0}{\max}h_{2}\left(V,V_{E},X_{3}\right)=\underset{X_{3}≽0}{\max}\log\left|X_{3}\right|-\mathrm{Tr}\left(X_{2}E_{3}(V,V_{E})\right)+N_{E}.$$

By introducing the slack variable $X_{3}\in {\mathbb{C}}^{N_{E}\times N_{E}}$, the MSE matrix of $f_{3}$ is

(30) $$E_{3}(V,V_{E})=I_{N_{E}}+H_{E}\left(VV^{H}+V_{E}V_{E}^{H}\right)H_{E}^{H},$$

Then the optimal $X_{3}$ can be expressed as

(31) $$X_{3}^{*}=\arg\underset{X_{3}≽0}{\max}h_{3}\left(V,V_{E},X_{3}\right)={\left[E_{3}(V,V_{E})\right]}^{-1}={\left[I_{N_{E}}+H_{E}\left(VV^{H}+V_{E}V_{E}^{H}\right)H_{E}^{H}\right]}^{-1}.\;$$

Therefore, the optimization problem can be expressed as

(32) $$\begin{array}{l} (P1):\;\underset{Z_{1},X_{1}≽0,Z_{2},X_{2}≽0,X_{3}≽0}{\max}R_{\sec}\\ \;=h_{1}\left(Z_{1},V,V_{E},X_{1}\right)+h_{2}\left(Z_{2},V_{E},X_{2}\right)+h_{3}\left(V,V_{E},X_{3}\right)\\ \;s.t.\;\mathrm{Tr}\left(VV^{H}+V_{E}V_{E}^{H}\right)\leq P_{\max}\\ \;\left|\theta_{m}\right|=1,\forall m\in M\\ \;\left\|p_{A}(n)-p_{A}(n-1)\right\|\leq \delta ,\forall n\in N. \end{array}$$

We can solve this problem by applying the AO method. Firstly, in the following sub-problem (P2), fix the value of $\Theta ,p_{A}$ to optimize the linear decoding matrices $Z_{1},Z_{2}$, slack variables $X_{1},X_{2}$, TPC matrix V, and AN matrix $V_{E}$. Secondly, in the following sub-problem (P3), fix the value of $Z_{1},X_{1},Z_{2},X_{2},X_{3},V,V_{E},$ and $p_{A}$ to optimize the phase shift matrix Θ. And finally, in the following sub-problem (P4), given $Z_{1},X_{1},Z_{2},X_{2},X_{3},V,V_{E},$ and Θ, the position of the UAV base station can be solved by the SRG method.

### 3.1. Optimizing $Z_{1},X_{1},Z_{2},X_{2},X_{3},V,$ and $V_{E}$

Simplifying Formula (32), the sub-optimization problem (P2) can be abbreviated as

(33) $$\begin{array}{l} (P2):\;\underset{V≽0,V_{E}≽0}{\min}\mathrm{Tr}\left(X_{1}Z_{1}^{H}H_{B}VV^{H}H_{B}^{H}Z_{1}\right)+\mathrm{Tr}\left(X_{1}Z_{1}^{H}H_{B}V_{E}V_{E}^{H}H_{B}^{H}Z_{1}\right)\\ \;-\mathrm{Tr}\left(X_{1}Z_{1}^{H}H_{B}V\right)-\mathrm{Tr}\left(X_{1}V^{H}H_{B}^{H}Z_{1}\right)-\mathrm{Tr}\left(X_{2}Z_{2}^{H}H_{E}V_{E}\right)-\mathrm{Tr}\left(X_{2}V_{E}^{H}H_{E}^{H}Z_{2}\right)\\ \;+\mathrm{Tr}\left(X_{2}Z_{2}^{H}H_{E}V_{E}V_{E}^{H}H_{E}^{H}Z_{2}\right)+\mathrm{Tr}\left(X_{3}H_{E}VV^{H}H_{E}^{H}\right)+\mathrm{Tr}\left(X_{3}H_{E}V_{E}V_{E}^{H}H_{E}^{H}\right)\\ \;s.t.\mathrm{Tr}\left(VV^{H}\right)+\mathrm{Tr}\left(V_{E}V_{E}^{H}\right)\leq P_{T}. \end{array}$$

The sub-problem is a convex QCQP problem for V and $V_{E}$, but the computational complexity is high if solved directly using CVX. Therefore, the Lagrange dual method is used to solve it. The Lagrangian function of (P2) is

(34) $$\begin{array}{l} \;L_{1}\left(V,V_{E},\mu \right)\\ =\mathrm{Tr}\left(X_{1}Z_{1}^{H}H_{B}VV^{H}H_{B}^{H}Z_{1}\right)+\mathrm{Tr}\left(X_{1}Z_{1}^{H}H_{B}V_{E}V_{E}^{H}H_{B}^{H}Z_{1}\right)-\mathrm{Tr}\left(X_{1}Z_{1}^{H}H_{B}V\right)\\ -\mathrm{Tr}\left(X_{1}V^{H}H_{B}^{H}Z_{1}\right)-\mathrm{Tr}\left(X_{2}Z_{2}^{H}H_{E}V_{E}\right)-\mathrm{Tr}\left(X_{2}V_{E}^{H}H_{E}^{H}Z_{2}\right)+\mathrm{Tr}\left(X_{2}Z_{2}^{H}H_{E}V_{E}V_{E}^{H}H_{E}^{H}Z_{2}\right)\\ +\mathrm{Tr}\left(X_{3}H_{E}VV^{H}H_{E}^{H}\right)+\mathrm{Tr}\left(X_{3}H_{E}V_{E}V_{E}^{H}H_{E}^{H}\right)+\mu \left[\mathrm{Tr}\left(VV^{H}\right)+\mathrm{Tr}\left(V_{E}V_{E}^{H}\right)-p_{T}\right]. \end{array}$$

And its dual problem is

(35) $$\underset{\mu \geq 0}{\max}\underset{V≽0,V_{E}≽0}{\min}L_{1}\left(V,V_{E},\mu \right).$$

By taking the first-order derivative, the optimal solutions of V and $V_{E}$ are obtained as

(36) $$\begin{array}{l} V^{*}={\left(\mu I_{N_{T}}+H_{V}\right)}^{†}H_{B}^{H}Z_{1}X_{1}^{H},\\ V_{E}^{*}={\left(\mu I_{N_{T}}+H_{VE}\right)}^{†}H_{E}^{H}Z_{2}X_{2}^{H}. \end{array}$$

where $H_{VE}=H_{B}^{H}Z_{1}X_{1}Z_{1}^{H}H_{B}+H_{E}^{H}Z_{2}X_{2}Z_{2}^{H}H_{E}+H_{E}^{H}X_{3}H_{E}$ and $H_{V}=H_{B}^{H}Z_{1}X_{1}Z_{1}^{H}H_{B}+H_{E}^{H}X_{3}H_{E}$.

Further, the eigenvalue decomposition is performed, and the optimal solution are written as

(37) $$\begin{array}{l} V^{*}=Q^{†}{\left(\mu I_{N_{T}}+\Lambda \right)}^{†}Q^{†}{}^{H}H_{B}^{H}Z_{1}X_{1}^{H},\\ V_{E}^{*}=Q_{E}{}^{†}{\left(\mu I_{N_{T}}+\Lambda_{E}\right)}^{†}Q_{E}^{†}{}^{H}H_{E}^{H}Z_{2}X_{2}^{H}. \end{array}$$

In addition, the optimal dual variable μ should satisfy

(38) $$\mu \left[\mathrm{Tr}\left(V^{*}V^{*}{}^{H}\right)+\mathrm{Tr}\left(V_{E}^{*}V^{*}{}_{E}^{H}\right)-P_{T}\right]=0.$$

Define the monotone non-increasing function $f\left(\mu \right)=$ $\mathrm{Tr}\left(VV^{H}\right)+\mathrm{Tr}\left(V_{E}V_{E}^{H}\right)$, we first check whether $f\left(0\right)\leq P_{\max}$ is satisfied when μ=0, if this condition is met, then $\mu^{*}=0$. Otherwise, the optimal value of the dual variable $\mu^{*}$ can be obtained by the bisection search method.

### 3.2. Optimizing Phase Shift Matrix

In this part, by fixing the value of $Z_{1},X_{1},Z_{2},X_{2},X_{3},V,V_{E},$ and $p_{A}$, the sub-optimization problem (P3) can be abbreviated as

(39) $$\begin{array}{l} (P3):\underset{V≽0,V_{E}≽0}{\min}\mathrm{Tr}\left(X_{1}Z_{1}^{H}H_{B}VV^{H}H_{B}^{H}Z_{1}\right)+\mathrm{Tr}\left(X_{1}Z_{1}^{H}H_{B}V_{E}V_{E}^{H}H_{B}^{H}Z_{1}\right)-\mathrm{Tr}\left(X_{1}Z_{1}^{H}H_{B}V\right)\\ \;-\mathrm{Tr}\left(X_{1}VH_{B}Z_{1}\right)-\mathrm{Tr}\left(X_{2}Z_{2}^{H}H_{E}V_{E}\right)-\mathrm{Tr}\left(X_{2}V_{E}^{H}H_{E}^{H}Z_{2}\right)\\ \;+\mathrm{Tr}\left(X_{2}Z_{2}^{H}H_{E}V_{E}V_{E}^{H}H_{E}^{H}Z_{2}\right)+\mathrm{Tr}\left(X_{3}H_{E}VV^{H}H_{E}^{H}\right)+\mathrm{Tr}\left(X_{3}H_{E}V_{E}V_{E}^{H}H_{E}^{H}\right)\\ \;s.t.\;\left|\theta_{m}\right|=1,\forall m\in M. \end{array}$$

Extracting Θ and $\Theta^{H}$ in the trace operation, the objective function of (P3) can be formulated as

(40) $$\mathrm{Tr}\left(\Theta^{H}D^{H}\right)+\mathrm{Tr}\left(\Theta D\right)+\mathrm{Tr}\left(\Theta F_{2}\Theta^{H}G_{2}\right)+\mathrm{Tr}\left(\Theta^{H}F_{1}\Theta G_{1}\right)+c_{t},$$

where $c_{t}$ is a constant term, which can be ignored in optimization, and

(41) $$V_{S}=VV^{H}+V_{E}V_{E}^{H},$$

(42) $$C_{1}=Z_{1}X_{1}Z_{1}^{H},$$

(43) $$C_{2}=Z_{2}X_{2}Z_{2}^{H},$$

(44) $$\begin{array}{ll} D & =H_{AR}V_{S}H_{AB}^{}C_{1}H_{RB}^{H}+H_{AR}V_{S}H_{AE}^{}X_{3}H_{RE}^{H}+H_{AR}V_{E}V_{E}^{H}H_{AE}^{}C_{2}H_{RE}^{H}\\ & -H_{AR}VX_{1}Z_{1}^{H}H_{RB}^{H}-H_{AR}V_{E}X_{2}Z_{2}^{H}H_{RE}^{H}, \end{array}$$

(45) $$G_{1}=H_{AR}VV_{}^{H}H_{AR}^{H},$$

(46) $$G_{2}=H_{AR}V_{E}V_{E}^{H}H_{AR}^{H},$$

(47) $$F_{1}=H_{RB}^{}C_{1}H_{RB}^{H}+H_{RE}^{}X_{3}H_{RE}^{H},$$

(48) $$F_{2}=F_{1}+H_{RE}^{}C_{2}H_{RE}^{H}.$$

By the Equation (1.9.5) in [35], remove the trace operation in the third and fourth terms of (40), namely:

(49) $$\mathrm{Tr}\left(\Theta^{H}F_{1}\Theta G_{1}\right)=\theta^{H}\left(F_{1}⊙G_{1}^{T}\right)\theta ,$$

(50) $$\mathrm{Tr}\left(\Theta^{H}F_{2}\Theta G_{2}\right)=\theta^{H}\left(F_{2}⊙G_{2}^{T}\right)\theta ,$$

where $\theta =[\theta_{1},\theta_{2},\ldots ,\theta_{M}]$, similarly, the trace operation in the first and second terms of (40) can be removed as

(51) $$\mathrm{Tr}\left(\Theta^{H}D^{H}\right)=d^{H}\left(\theta^{*}\right)\Theta \mathrm{Tr}\left(\Theta D\right)=\theta^{T}d,$$

where $\theta^{*}$ is the conjugate vector of θ and d is the vector composed of elements on the diagonal of D. Thus, the phase shift matrix optimization problem can be rewritten as

(52) $$\begin{array}{l} \underset{\phi }{\min}f\left(\theta \right)=\theta^{H}\Xi \theta +\theta^{T}d+d^{H}\theta^{*}\\ s.t.\left|\theta_{m}\right|=1,\forall m\in M, \end{array}$$

where $\Xi =F_{2}⊙G_{2}^{T}+F_{1}⊙G_{1}^{T}$. The right side of the equation is positive semi-definite matrices, so Ξ is also a positive semi-definite matrix. Then, the optimization problem simplifies to

(53) $$\begin{array}{l} \underset{\phi }{\min}f\left(\theta \right)=\theta^{H}\Xi \theta +2ℜ\left\{\theta^{H}d^{*}\right\}\\ s.t.\left|\theta_{m}\right|=1,\forall m\in M. \end{array}$$

For the form of the optimization problem in (53), we solve it using three low complexity methods: ADMM [36], MM [37], and RCG [38].

#### 3.2.1. Alternating Direction Method of Multipliers

Introducing an auxiliary variable q for θ, then the optimization problem can be reformulated as

(54) $$\begin{array}{l} \underset{\theta ,q}{\max}\;{\hat{f}}_{1}\left(\theta \right)=-\theta^{H}\Xi \theta -2ℜ\left\{\theta^{H}d^{*}\right\}-\frac{\xi }{2}{\left\|q-\theta \right\|}_{2}^{2}\\ \;s.t.q=\theta \\ \;\left|\theta_{m}\right|=1,\forall m\in M, \end{array}$$

where ξ>0 is the penalty parameter. Defining $g_{2}\left(\theta_{m}\right)=0$ if $\left|\theta_{m}\right|=1$ and $g_{2}\left(\theta_{m}\right)=\infty$, if $\left|\theta_{m}\right|\neq 1$, $\rho_{R}={\left[\rho_{R,1},\ldots ,\rho_{R,1}\right]}^{T}$ and $\rho_{I}={\left[\rho_{I,1},\ldots ,\rho_{I,1}\right]}^{T}$ are Lagrange variables corresponding to the real and imaginary parts of q−θ=0, respectively. Let $\rho =\rho_{R}+\rho_{I}$, then its Lagrange function can be written as

(55) $$L_{2}\left(q,\theta ,\lambda_{R},\lambda_{I}\right)=-q^{H}\Xi q-\sum_{m=1}^{M}g_{1}\left(\theta_{m}\right)-\frac{\xi }{2}{\left\|q-\theta \right\|}_{2}^{2}+ℜ\left\{-2q^{H}d^{*}+{\left(\rho_{R}+j\rho_{I}\right)}^{H}\left(q-\theta \right)\right\}.$$

Its dual function is

(56) $$\underset{\rho_{R},\rho_{I}}{\min}\;\;G\left(\rho_{R},\rho_{I}\right)=\underset{q,\theta }{\max}L_{2}\left(q,\theta ,\rho_{R},\rho_{I}\right).$$

Therefore, the iterative form of ADMM solution for the dual problem is

(57) $$\theta^{\left(n+1\right)}=\arg\underset{\theta }{\max}L_{2}\left(q^{\left(n\right)},\theta ,{\bar{\rho }}^{\left(n\right)}\right),$$

(58) $$q^{\left(n+1\right)}=\arg\underset{q}{\max}L_{2}\left(q,\theta^{\left(n+1\right)},{\bar{\rho }}^{\left(n\right)}\right),$$

(59) $${\bar{\rho }}^{\left(n+1\right)}={\bar{\rho }}^{\left(n\right)}-\xi \left(q^{\left(n+1\right)}-\theta^{\left(n+1\right)}\right).$$

For the iteration of $q^{\left(n+1\right)}$, it is necessary to derive the objective function, and if the derivative is zero, the specific closed expression can be obtained as

(60) $$q^{\left(n+1\right)}={\left(2\Xi +\xi I_{M}\right)}^{-1}\left(-2d^{*}+{\bar{\rho }}^{\left(n\right)}+\xi \theta^{\left(n+1\right)}\right).$$

For the iteration of $\theta^{\left(n+1\right)}$, taking out the terms related to θ in $L_{2}$, the optimization problem can be written as

(61) $$\underset{\theta }{\max}-\frac{\xi }{2}{\left\|q^{\left(n\right)}-\theta \right\|}_{2}^{2}+ℜ\left\{{\left(\rho_{R}^{\left(n\right)}+j\rho_{I}^{\left(n\right)}\right)}^{H}\left(q^{\left(n\right)}-\theta \right)\right\}-\sum_{m=1}^{M}g_{1}\left(\theta_{m}\right).$$

Since the constant term does not affect the solution of the optimization problem, we continue to add the constant term and express the objective function as

(62) $$\underset{\theta }{\max}-\frac{\xi }{2}{\left\|q^{\left(n\right)}-\frac{1}{\xi }{\bar{\rho }}^{\left(n\right)}-\theta \right\|}_{2}^{2}-\sum_{m=1}^{M}g_{1}\left(\theta_{m}\right).$$

If we want to obtain the maximum value of the objective function, $\theta^{\left(n+1\right)}$ needs to satisfy

(63) $$\theta^{\left(n+1\right)}=\mathrm{Pj}(q^{\left(n\right)}-\frac{1}{\xi }{\bar{\rho }}^{\left(n\right)}).$$

Let $\bar{\theta }=q^{\left(n\right)}-{\bar{\rho }}^{\left(n\right)}/\xi$, then the projection operation can be expressed as

(64) $$∠\theta_{m}^{\left(n+1\right)}=∠{\bar{\theta }}_{m}^{}.$$

According to Lemma 3 in [36], we choose $\xi =\iota {\left\|\Xi \right\|}_{2}$, where ι≥1 is the minimum integer which satisfies $(\xi /2)I_{M}-\Xi ≻0$.

#### 3.2.2. Majorization-Minimization

The core idea of the MM algorithm is to design a series of approximate optimization functions to control the upper limit of the original function, and to converge to the optimal solution of the original objective by minimizing the sequence.

We use ${\hat{f}}_{1}\left(\theta \right)$ to represent the upper bound of the objective function. According to Lemma 1 in [37], we reformulate the problem and rewritten as

(65) $$\begin{array}{l} \min{\hat{f}}_{2}\left(\theta \right)=g_{2}\left(\theta \right)+2ℜ\left\{\theta^{H}d^{*}\right\}\\ s.t.\left|\theta_{m}\right|=1,\forall m\in M. \end{array}$$

Since $\theta^{H}\theta =M$, $\theta^{H}\lambda_{\max}I_{M}\theta =M\lambda_{\max}$ is constant, after removing the constant term, the sub-problem becomes

(66) $$\begin{array}{l} \min-2ℜ\left\{\theta^{H}\left(\left(\lambda_{\max}I_{M}-\Xi \right)\theta^{\left(n\right)}-d^{*}\right)\right\}\\ s.t.\left|\theta_{m}\right|=1,\forall m\in M. \end{array}$$

Therefore, its optimal solution is given by

(67) $$\theta^{\left(n+1\right)}=\exp\left(j\arg\left[\left(\lambda_{\max}I_{M}-\Xi \right)\theta^{\left(n\right)}-d^{*}\right]\right),$$

where $\lambda_{\max}$ is the maximum eigenvalue of Ξ.

#### 3.2.3. Riemannian Conjugate Gradient

The RCG method has been widely used in IRS-assisted MISO and MU-MISO communications, so this problem can also be solved using the RCG method. The specific steps are as follows.

1. Compute Riemannian Gradient: Based on the manifold space constrained by the IRS phase shift matrix, we first calculate the Riemann gradient as the orthogonal projection of the Euclidean gradient on the tangent space,
  (68) $$\mathrm{grad}f\left(\theta^{\left(n\right)}\right)=\nabla f\left(\theta^{\left(n\right)}\right)-ℜ\left\{\nabla f\left(\theta^{\left(n\right)}\right)⊙\theta^{\left(n\right)}{}^{*}\right\}⊙\theta^{\left(n\right)},$$
  where $\nabla \hat{f}\left(\theta^{\left(n\right)}\right)=2\Xi \theta^{\left(n\right)}+2d^{*}$.
2. Search Direction: The conjugate search direction on the tangent space is
  (69) $$r^{\left(n+1\right)}=-\mathrm{grad}f\left(\theta^{\left(n\right)}\right)+\zeta_{1}Ζ\left(r^{\left(n\right)}\right),$$
  where Ζ(⋅) is the vector transport function, and it can be expressed as
  (70) $$Ζ\left(r^{\left(n\right)}\right)=r^{\left(n\right)}-ℜ\left\{r^{\left(n\right)}⊙\theta^{\left(n\right)}{}^{*}\right\}⊙\theta^{\left(n\right)},$$
  where $\zeta_{1}$ is the Polak-Ribière parameter [39].
3. Retraction: Project the tangent vector back to the circular manifold,
  (71) $$\theta^{\left(n+1\right)}=\mathrm{Pj}⟮\frac{\theta^{\left(n\right)}+\zeta_{2}r^{\left(n+1\right)}}{\left|\theta^{\left(n\right)}+\zeta_{2}r^{\left(n+1\right)}\right|}⟯,$$
  where $\zeta_{2}$ denotes the Armijo backtracking line step size [39].

### 3.3. Optimizing UAV Placement

Aiming at the influence of UAV on the spectral efficiency of centralized radio access network, Roth et al. proposed a UAV location optimization method to maximize the data rate [40], but this method does not consider the influence of IRS and eavesdroppers. Based on this work, in this part, we propose a secure position searching method for IRS-assisted UAV-MIMO systems–namely the SRG method. The details are as follows.

Fixing other variables except $p_{A}$, the sub-optimization problem of UAV placement (P4) can be expressed as

(72) $$\begin{array}{l} \left(P4\right):\;\underset{p_{A}}{\max}R_{\sec}=\log_{2}\left|I_{N_{B}}+H_{B}VV^{H}H_{B}^{H}J_{B}^{-1}\right|-\log_{2}\left|I_{N_{E}}+H_{E}VV^{H}H_{E}^{H}J_{E}^{-1}\right|\\ \;s.t.\left\|p_{A}(n)-p_{A}(n-1)\right\|\leq \delta ,\forall n\in N. \end{array}$$

The SRG method requires the derivative of the SR $R_{\sec}$ with respect to the UAV coordinate, according to [41]

(73) $$\begin{array}{l} \frac{\partial R_{\sec}}{\partial p_{A,\gamma }}=\frac{1}{\ln2}\cdot \mathrm{Tr}\left\{{\left(I_{N_{B}}+H_{B}VV^{H}H_{B}^{H}J_{B}^{-1}\right)}^{-1}\cdot \frac{\partial H_{B}VV^{H}H_{B}^{H}J_{B}^{-1}}{\partial p_{A,\gamma }}\right\}\\ -\frac{1}{\ln2}\cdot \mathrm{Tr}\left\{{\left(I_{N_{B}}+H_{E}VV^{H}H_{E}^{H}J_{E}^{-1}\right)}^{-1}\cdot \frac{\partial H_{E}VV^{H}H_{E}^{H}J_{E}^{-1}}{\partial p_{A,\gamma }}\right\}, \end{array}$$

where the derivative of $H_{B}$ with respect to $p_{A,\gamma }^{}$ can be expressed as

(74) $$\begin{array}{cc} \; & \frac{\partial H_{B}}{\partial p_{A,\gamma }}=\frac{\sqrt{\beta_{0}}}{\sigma_{B}^{}}\frac{\partial }{\partial p_{A,\gamma }}\frac{1}{d_{AR}^{c_{AR}/2\;}}H_{RB}^{T}\Theta {\tilde{H}}_{AR}+\frac{\sqrt{\beta_{0}}}{\sigma_{B}^{}}\frac{\partial }{\partial p_{A,\gamma }}\frac{1}{d_{AB}^{c_{AB}/2\;}}{\tilde{H}}_{AB}^{T}\\ \; & \;=\frac{\sqrt{\beta_{0}}}{\sigma_{B}^{}}\cdot \frac{c_{AR}}{2}\frac{\left(p_{R,\gamma }^{}-p_{A,\gamma }^{}\right)}{{∥p_{R}^{}-p_{A}^{}∥}^{c_{AR}/2\;+2}}H_{RB}^{T}\Theta {\tilde{H}}_{AR}+\frac{\sqrt{\beta_{0}}}{\sigma_{B}^{}{∥p_{R}^{}-p_{A}^{}∥}^{c_{AR}/2\;}}H_{RB}^{T}\Theta \frac{\partial {\tilde{H}}_{AR}}{\partial p_{A,\gamma }}\\ \; & \;+\frac{\sqrt{\beta_{0}}}{\sigma_{B}^{}}\cdot \frac{c_{AB}}{2}\frac{\left(p_{B,\gamma }^{}-p_{A,\gamma }^{}\right)}{{∥p_{B}^{}-p_{A}^{}∥}^{c_{AB}/2\;+2}}{\tilde{H}}_{AB}^{T}+\frac{\sqrt{\beta_{0}}}{\sigma_{B}^{}{∥p_{B}^{}-p_{A}^{}∥}^{c_{AB}/2\;}}\frac{\partial {\tilde{H}}_{AB}^{T}}{\partial p_{A,\gamma }}. \end{array}$$

Similarly, the derivative of $H_{E}$ with respect to $p_{A,\gamma }^{}$ can be expressed as

(75) $$\begin{array}{cc} \; & \frac{\partial H_{E}}{\partial p_{\gamma }}\approx \frac{\sqrt{\beta_{0}}}{\sigma_{E}^{}}\frac{\partial }{\partial p_{A,\gamma }}\frac{1}{d_{AR}^{c_{AR}/2\;}}H_{RE}^{T}\Theta {\tilde{H}}_{AR}+\frac{\sqrt{\beta_{0}}}{\sigma_{E}^{}}\frac{\partial }{\partial p_{A,\gamma }}\frac{1}{d_{AE}^{c_{AE}/2\;}}{\tilde{H}}_{AE}^{T}\\ \; & \;=\frac{\sqrt{\beta_{0}}}{\sigma_{E}^{}}\frac{c_{AR}}{2}\frac{\left(p_{R,\gamma }^{}-p_{A,\gamma }^{}\right)}{{∥p_{R}^{}-p_{A}^{}∥}^{c_{AR}/2\;+2}}H_{RE}^{T}\Theta {\tilde{H}}_{AR}+\frac{\sqrt{\beta_{0}}}{\sigma_{E}^{}{∥p_{R}^{}-p_{A}^{}∥}^{c_{AR}/2\;}}H_{RE}^{T}\Theta \frac{\partial {\tilde{H}}_{AR}}{\partial p_{A,\gamma }}\\ \; & \;+\frac{\sqrt{\beta_{0}}}{\sigma_{E}^{}}\frac{c_{AE}}{2}\frac{\left(p_{E,\gamma }^{}-p_{A,\gamma }^{}\right)}{{∥p_{E}^{}-p_{A}^{}∥}^{c_{AE}/2\;+2}}{\tilde{H}}_{AE}^{T}+\frac{\sqrt{\beta_{0}}}{\sigma_{E}^{}{∥p_{E}^{}-p_{A}^{}∥}^{c_{AE}/2\;}}\frac{\partial {\tilde{H}}_{AE}^{T}}{\partial p_{A,\gamma }}. \end{array}$$

In the previous research on position optimization and trajectory planning of UAV communication, it was often assumed that the influence of the phase part on the channel was negligible and focused on the influence of distance change on the channel. In this case, we also assume that the phase only has some random and subtle influence on the channel. Therefore, the influence of this part on the position change can be ignored. The derivative part in (73) can be expressed as

(76) $$\begin{array}{cc} \; & \;\frac{\partial H_{B}VV^{H}H_{B}^{H}J_{B}^{-1}}{\partial p_{A,\gamma }}=\frac{\partial H_{B}VV^{H}H_{B}^{H}J_{B}^{-1}}{\partial H_{B}}\cdot \frac{\partial H_{B}}{\partial p_{A,\gamma }}\\ \; & \approx ⟮\frac{c_{AR}}{2\sigma_{B}}\frac{\left(p_{R,\gamma }^{}-p_{A,\gamma }^{}\right)}{{∥p_{R}^{}-p_{A}^{}∥}^{2}}H_{RB}^{T}\Theta H_{AR}+\frac{c_{AB}}{2\sigma_{B}}\frac{\left(p_{B,\gamma }^{}-p_{A,\gamma }^{}\right)}{{∥p_{B}^{}-p_{A}^{}∥}^{2}}H_{AB}^{T}⟯VV^{H}H_{B}^{H}J_{B}^{-1}\\ \; & +H_{B}^{}VV^{H}{⟮\frac{c_{AR}}{2\sigma_{B}}\frac{\left(p_{R,\gamma }^{}-p_{A,\gamma }^{}\right)}{{∥p_{R}^{}-p_{A}^{}∥}^{2}}H_{RB}^{T}\Theta H_{AR}+\frac{c_{AB}}{2\sigma_{B}}\frac{\left(p_{B,\gamma }^{}-p_{A,\gamma }^{}\right)}{{∥p_{B}^{}-p_{A}^{}∥}^{2}}H_{AB}^{T}⟯}^{H}J_{B}^{-1}, \end{array}$$

(77) $$\begin{array}{cc} \; & \;\frac{\partial H_{E}VV^{H}H_{E}^{H}J_{E}^{-1}}{\partial p_{A,\gamma }}=\frac{\partial H_{E}VV^{H}H_{I}^{H}J_{E}^{-1}}{\partial H_{E}}\cdot \frac{\partial H_{E}}{\partial p_{A,\gamma }}\\ \; & \approx ⟮\frac{c_{AR}}{2\sigma_{E}}\frac{\left(p_{R,\gamma }^{}-p_{A,\gamma }^{}\right)}{{∥p_{R}^{}-p_{A}^{}∥}^{2}}H_{RE}^{T}\Theta H_{AR}+\frac{c_{AE}}{2\sigma_{E}}\frac{\left(p_{E,\gamma }^{}-p_{A,\gamma }^{}\right)}{{∥p_{E}^{}-p_{A}^{}∥}^{2}}H_{AE}^{T}⟯VV^{H}H_{E}^{H}J_{E}^{-1}\\ \; & +H_{E}^{}VV^{H}{⟮\frac{c_{AR}}{2\sigma_{E}}\frac{\left(p_{R,\gamma }^{}-p_{A,\gamma }^{}\right)}{{∥p_{R}^{}-p_{A}^{}∥}^{2}}H_{RE}^{T}\Theta H_{AR}+\frac{c_{AE}}{2\sigma_{E}}\frac{\left(p_{E,\gamma }^{}-p_{A,\gamma }^{}\right)}{{∥p_{E}^{}-p_{A}^{}∥}^{2}}H_{AE}^{T}⟯}^{H}J_{E}^{-1}\;. \end{array}$$

We can obtain the gradient of SR as

(78) $${\left(\nabla R_{\sec}\right)}_{\gamma }=\frac{\partial R_{\sec}}{\partial p_{A,\gamma }}.$$

Next, the state of the UAV is adjusted and controlled by the secrecy rate gradient. When the UAV achieves the maximum SR at a certain point, the position of the UAV should remain unchanged; when the secrecy rate gradient is not zero, the UAV should move along the gradient direction.

**Figure 2:**
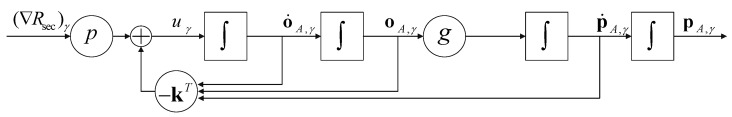
The feedback control process of UAV systems based on PI-controller.

In order to meet the above requirements, the static end value of ${\dot{p}}_{A}^{}$ should match the derivative $\nabla R_{\sec}$ of constant SR. So, with the PI-controller, the input signal is designed as

(79) $$u_{\gamma }=p{\left(\nabla R_{\sec}\right)}_{\gamma }-k^{T}⟮\begin{array}{c} {\dot{p}}_{A,\gamma }^{\left(n\right)}\\ o_{A,\gamma }^{\left(n\right)}\\ {\dot{o}}_{A,\gamma }^{\left(n\right)} \end{array}⟯,\forall \gamma \in \{1,2\},$$

where $k={\left(\begin{array}{ccc} k_{1} & k_{2} & k_{3} \end{array}\right)}^{T}$ is the controller gain, p is the prefilter coefficient, and the system feedback control flow based on the PI-controller is shown in Figure 2, where the ⊕ represents the superposition of signals, which are calculated by the process indicated by arrows.

In the control system, the controller matrices are determined by a linear quadratic regulator (LQR). Then, the parameters of the whole control system can be deduced as follows:

(80) $$A=⟮\begin{array}{cccc} 0 & 1 & 0 & 0\\ 0 & 0 & 1 & 0\\ 0 & 0 & 0 & 1\\ 0 & -k_{1} & -k_{2} & -k_{3} \end{array}⟯,b=⟮\begin{array}{c} 0\\ 0\\ 0\\ p \end{array}⟯.$$

Therefore, the state equation can be evaluated by

(81) $$⟮\begin{array}{c} {\dot{c}}_{1}\\ {\dot{c}}_{2} \end{array}⟯=\left(I_{2}⊗A\right)⟮\begin{array}{c} c_{1}\\ c_{2} \end{array}⟯+⟮⟮\begin{array}{ccc} 1 & 0 & 0\\ 0 & 1 & 0 \end{array}⟯⊗b⟯\nabla R_{\sec}.$$

By superimposing the results obtained in the (81) with the state vector of the previous moment, the optimized position of the UAV can be finally obtained.

### 3.4. Overall Algorithm and Complexity Discussion

With the proposed optimization method of the three sub-problems, the overall algorithm for solving the problem (P1) is summarized in Algorithm 1. Specifically, we iteratively solve three sub-problems–(P2), (P3), and (P4)–using the alternating optimization method. For these three blocks, we fix the other two blocks to optimize a certain block. The solution of other sub-problems obtained in this iteration are used as input to solve the next sub-problem. When the change of SR obtained by two consecutive iterations is less than the convergence accuracy χ or the number of iterations exceeds $j^{\max}$, the iterative solution can be stopped. The TPC matrix $V^{0}$ and AN matrix $V_{E}{}^{0}$ are initialized as matrices whose internal elements are all $1/\sqrt{N_{d}}$ and $1/\sqrt{N_{T}}$, respectively. And the phase shift matrix $Q^{0}$ is initialized to the identity matrix.
**Algorithm 1** Iteration Algorithm for Optimizing Problem (P1) 
**Input**: $V^{0}$, $V_{E}{}^{0}$, $\Theta^{0}$, $p_{A}{}^{0}$, χ.
 
**Output**: the TPC matrix and AN matrix, the IRS phase shift matrices, the UAV position, and the SR.
 **Initialize**: the iterative number j=0.
 **Repeat**
 1: update $\left\{Z_{1}{}^{j+1},X_{1}{}^{j+1},Z_{2}{}^{j+1},X_{2}{}^{j+1},X_{3}{}^{j+1}\right\}$, and solve problem (P2) to obtain optimal $\left\{V^{j+1},V_{E}{}^{j+1}\right\}$ for given $\left\{\Theta^{j},p_{A}{}^{j}\right\}$.
 2: Solve problem (P3) to obtain the optimal $\left\{\Theta^{j+1}\right\}$ for given $\{Z_{1}{}^{j+1},X_{1}{}^{j+1},Z_{2}{}^{j+1},X_{2}{}^{j+1},$$X_{3}{}^{j+1},V^{j+1},V_{E}{}^{j+1},p_{A}{}^{j}\}$. When choosing to use the ADMM algorithm, iteratively solve by Equations (57)–(59); when choosing to use the MM algorithm, iteratively solve by Equation (67); when choosing to use the RCG algorithm, iteratively solve by Equations (69)–(71) until the SR increment or the maximum iteration number is reached to end the loop of this sub-problem.
 3: Solve problem (P4) to obtain the $\left\{p_{A}{}^{j+1}\right\}$ for given $\{Z_{1}{}^{j+1},X_{1}{}^{j+1},Z_{2}{}^{j+1},X_{2}{}^{j+1},X_{3}{}^{j+1},$$V^{j+1},V_{E}{}^{j+1},\Theta^{j+1}\}$. Using the SRG algorithm, iteratively solves Equation (81) until the SR increment or the maximum iteration number is reached to end the loop of this sub-problem.
4: Set j=j+1.
**until**  $\left|\left(R_{\sec,j}^{}-R_{\sec,j-1}^{}\right)/R_{\sec,j}^{}\right|\leq \chi$ or $j>j^{\max}$.

For the solution of the IRS phase shift matrix, the iterative process in the ADMM, MM, and RCG sub-algorithms can guarantee the monotonic descent of the sub-problem (P3). Similarly, for the solution of the UAV position, the iterative process in the SRG sub-algorithm also ensures the monotonic decline of the sub-problem (P4). For the solution process of the whole algorithm, the AO algorithm can guarantee the monotonic decrease of the objective function value in each iteration, and the existence of the transmit power constraint ensures the convergence of Algorithm 1.

The complexity of algorithm 1 is analyzed below. In step 1, the computational complexity of $\left\{H_{V},H_{VE}\right\}$ is $O(N_{T}^{3})+O(2N_{T}^{2}N_{d})+O(2N_{T}^{2}N_{E})$, the complexity of binary search to find the optimal μ is $O(N_{T}^{2}N_{B})+O(2N_{T}^{3})$, then the computational complexity $C_{2}$ of sub-problem (P2) is $O(\max\{2N_{T}^{3},2N_{T}^{2}N_{E}\})$. In step 2, the computational complexity $C_{3}$ of ADMM, MM, and RCG algorithm are $O(M_{}^{3}+T_{ADMM}M_{}^{3})$, $O(M_{}^{3}+T_{MM}M_{}^{2})$, and $O(M_{}^{3}+T_{RCG}M_{}^{2})$, respectively, where $T_{ADMM}$, $T_{MM}$, and $T_{RCG}$ represent the iteration number of three IRS shift optimization sub-algorithms. In step 3, the computational complexity $C_{4}$ of the SRG algorithm is $O(T_{SRG}N_{T}^{2})$, where $T_{SRG}$ represent the iteration number of the SRG algorithm. Therefore, the complexity of the whole problem can be presented by

(82) $$C_{1}=O(\max\{C_{2},C_{3},C_{4}\}).$$

## 4. Simulation Results

In this section, we show the simulation results to verify the effectiveness of Algorithm 1 and the advantage of the proposed secure transmission strategy. The parameter settings we used in the simulation are set as follows unless otherwise specified [31,40]. The coordinates of initial UAV point $p_{A}{}^{0}$, IRS, Bob, and Eve are set as ${\left[-120,1.5\right]}^{T}$, ${\left[0,0\right]}^{T}$, ${\left[10,15\right]}^{T}$, and ${\left[-10,15\right]}^{T}$, respectively. The height of UAV is 60 m and the height of IRS is 25 m. The transmit power limit is $P_{T}=15\;\mathrm{dBm}$. The antennas number of UAV, Bob, Eve, and IRS are $N_{T}=4$, $N_{B}=2$, $N_{E}=2$, and M=48, respectively. The number of data streams is set to $N_{d}=2$. Rician factor is set to $k_{ab}=4$. Path loss exponent $c_{AR}=2.2$; $c_{RB}$ and $c_{RE}$ are set to 2.5; $c_{AB}$ and $c_{AE}$ are set to 3.5; and the path loss at the reference distance 1 m is set to −30 dB. The Bob’s noise power and the Eve’s noise power are $\sigma_{B}^{2}=-75\;\mathrm{dBm}$ and $\sigma_{E}^{2}=-75\;\mathrm{dBm}$. The maximum UAV moving distance between two iterations is set to δ=0.6. The controller gain $k={\left(\begin{array}{ccc} 0.5477 & 23.9683 & 6.9308 \end{array}\right)}^{T}$. The prefilter coefficient p=0.0175. The initial UAV states vectors are set to $c_{\gamma ,0}={\left(\begin{array}{cccc} p_{A,\gamma }^{0} & 0 & 0 & 0 \end{array}\right)}^{T}$, ∀γ∈{1,2}. The convergence accuracy $\chi =10^{-5}$ and the maximum number of iterations $j^{\max}=10^{3}$.

**Figure 3:**
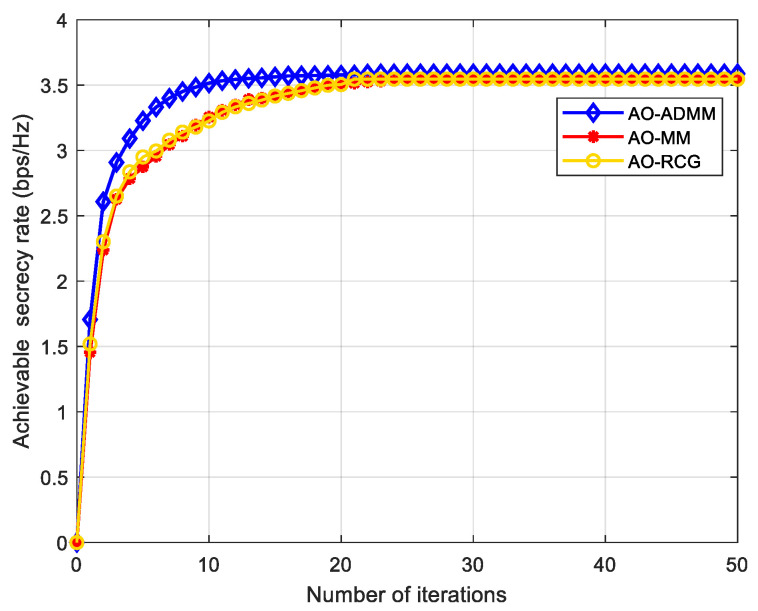
Convergence performance comparison with Algorithm 1.

The convergence performance of our proposed Algorithm 1 is described in Figure 3, where the ADMM, MM, and RCG algorithms are used to optimize the IRS phase shift matrix, respectively. It can be seen from Figure 3 that for the communication scenario in this paper, with the increase of the number of iterations, the SR gradually increases and converges at about 3.58 after 20~30 iterations, and the convergence speed and secrecy performance of the AO-ADMM algorithm has a little advantage over other two algorithms.

**Figure 4:**
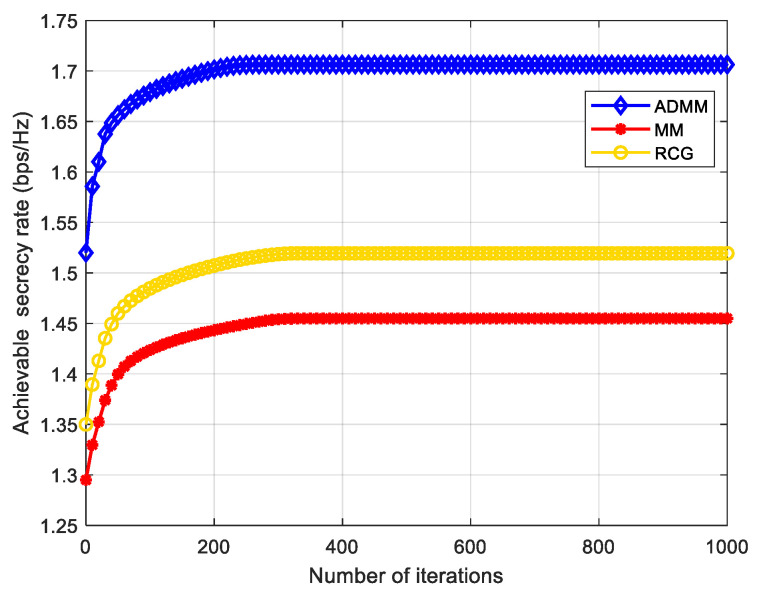
Convergence performance comparison of the ADMM, MM, and RCG Algorithm.

In each iteration of Algorithm 1, ADMM, MM, and RCG are used to solve the problem (P3), the phase shift matrix of IRS. Figure 4 shows the convergence performance of the three algorithms in the first iteration of our proposed AO algorithm. As can be seen from Figure 4, the convergence speed of the three algorithms is slightly different, but not by much. Based on the analysis in Section 3, the computational complexity of MM and RCG algorithms is similar, and the computational complexity of the ADMM algorithm is higher than the other two algorithms. Although the computational complexity of the AO-ADMM algorithm is higher, its convergence performance and SR are slightly better than the other two algorithms. Therefore, we use the AO-ADMM algorithm to compare the performance with other benchmark schemes in the following simulation experiments.

**Figure 5:**
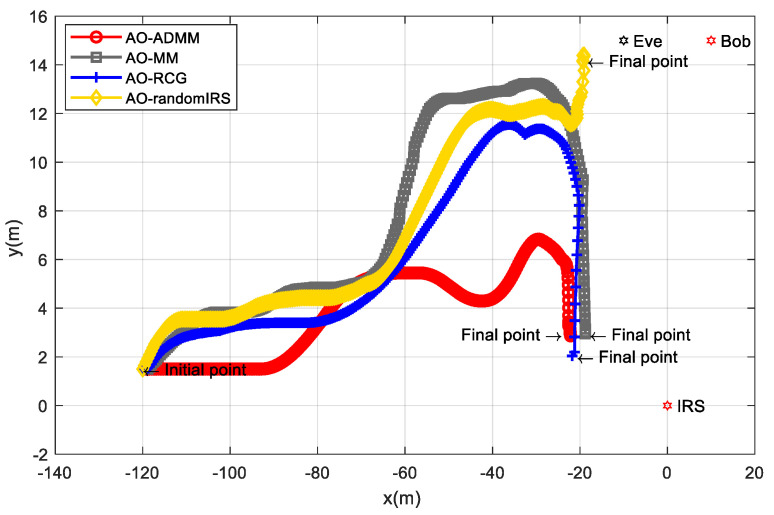
The UAV position change process with different IRS optimization method.

Figure 5 shows the UAV position change process of the AO algorithms based on three different phase shift matrix optimization methods. As a comparison, we also introduce an AO algorithm with a randomly generated IRS phase shift matrix. It can be seen from Figure 5 that for the case of randomly setting the IRS phase shift, the UAV will move towards the nearby position of the receiver and the eavesdropper. The UAV will not be completely close to Eve and Bob and will finally stop at the ${\left[-19.34,14.17\right]}^{T}$. This is because although the phase shift matrix is not optimal in this case, the IRS can still play a positive role in improving the secrecy performance. For the case of adopting ADMM, MM, and RCG algorithms, the UAV will continue to turn to the nearby position where the IRS is located, and finally stopped at ${\left[-22.23,2.95\right]}^{T}$, ${\left[-18.81,2.94\right]}^{T}$, and ${\left[-21.79,2.04\right]}^{T}$, respectively. This is because the optimized IRS can give a full play to its secrecy ability, and the UAV can obtain a higher SR in the direction close to the IRS, so the UAV will move closer to the IRS. That is to say, the proposed SRG position optimization algorithm and three IRS phase shift matrix optimization methods are important means to effectively improve the secrecy ability.

Next, we consider different benchmark schemes to verify the advantages of our proposed method. The different schemes are shown as follows.

Scheme 1: the TPC matrix, AN matrix, phase shift matrix, and position are optimized via the proposed AO-ADMM algorithm.

Scheme 2: The same as Scheme 1 except that the phase shift matrix is set randomly.

Scheme 3: The same as Scheme 1 except that the TPC matrix is set randomly.

Scheme 4: The same as Scheme 1 except that the AN matrix is set randomly.

Scheme 5: The same as Scheme 1 except that the IRS phase shift matrix is optimized by the one-by-one (OBO) algorithm in paper [42].

**Figure 6:**
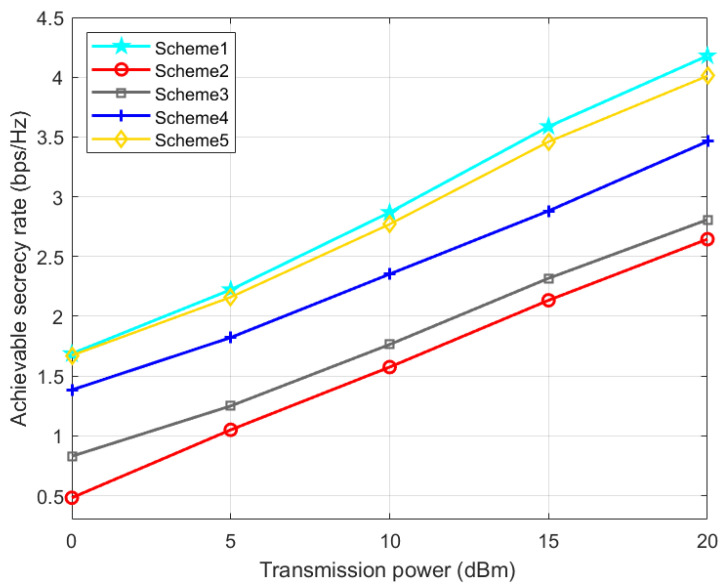
Achievable SR versus the transmission power.

It can be seen from Figure 6 that as the UAV base station transmission power increases, the achievable SR of all five schemes will increase. Moreover, scheme 1 using the ADMM algorithm is always better than scheme 5 using the OBO algorithm for IRS optimization.

**Figure 7:**
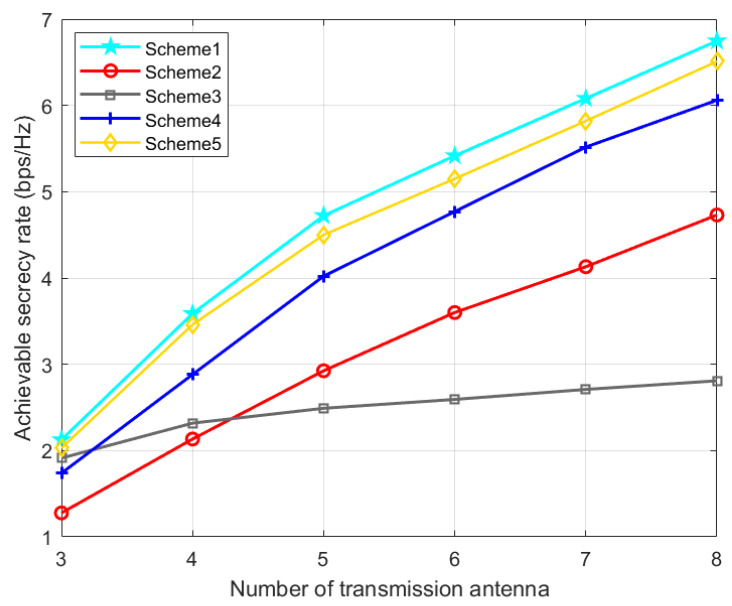
Achievable SR versus the number of transmission antenna.

As can be seen from Figure 7, with the increase in the number of transmission antennas, the achievable SR of all five schemes also increases. In Figure 7, in the whole region, our proposed scheme 1 has the highest achievable rate. When the number of transmission array is small, compared with scheme 4 and scheme 2, scheme 3 has better confidentiality ability. In addition, when the number of array elements is large, the SR of scheme 4 and scheme 2 is better than that of scheme 3. This is because the number of transmit antennas has a great influence on the performance of TPC matrices.

**Figure 8:**
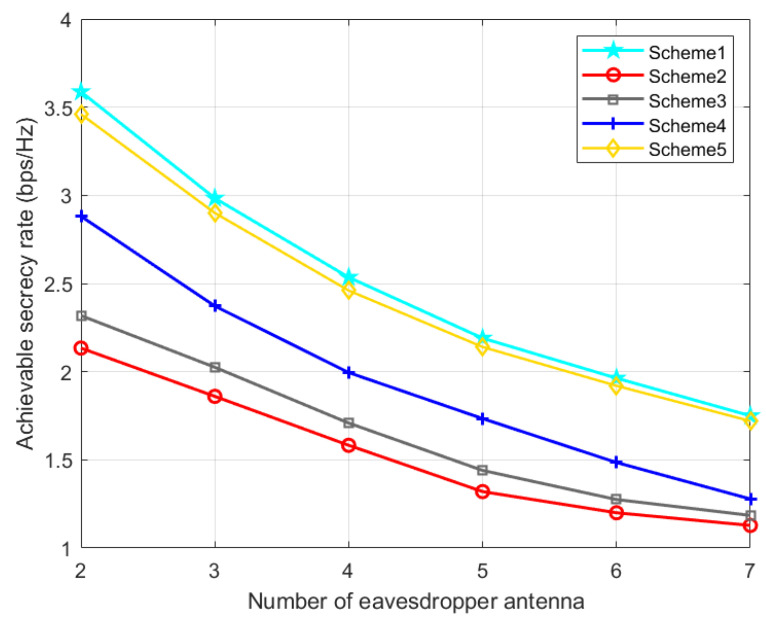
Achievable SR versus the number of eavesdropper antenna.

The effect of the number of eavesdropper antenna on the secrecy performance is described in Figure 8. As the number of eavesdropper antennas increases, the SR of all schemes will decline, and our proposed optimization method is always better than several other schemes.

**Figure 9:**
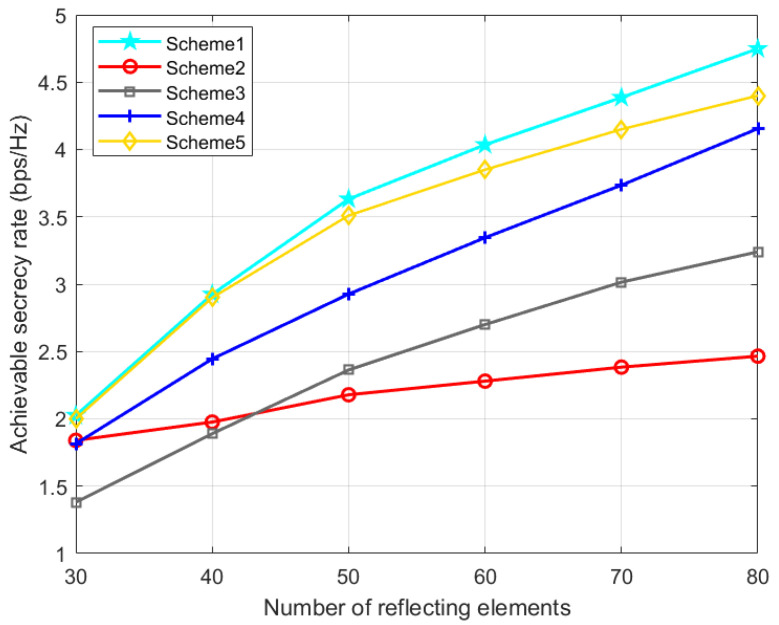
Achievable SR versus the number of reflecting elements.

Figure 9 shows the impact of the IRS elements number on the secrecy performance of the UAV system. It is observed from Figure 9 that with the increase in the number of reflection elements, the SR of all schemes will increase. However, due to the lack of optimization of the phase shift matrix in scheme 2, the SR increases very slowly, which shows the importance of IRS in our proposed joint optimization algorithm. With the preset parameter settings, the SR of our proposed scheme 1 can be about 40.5% higher than that of scheme 2 without IRS. Compared with Scheme 5 using the OBO algorithm, the performance advantage of our proposed Scheme 1 becomes more obvious as the number of IRS elements increases. It proves the effectiveness of Algorithm 1 and the significance of IRS in secure UAV communication.

**Figure 10:**
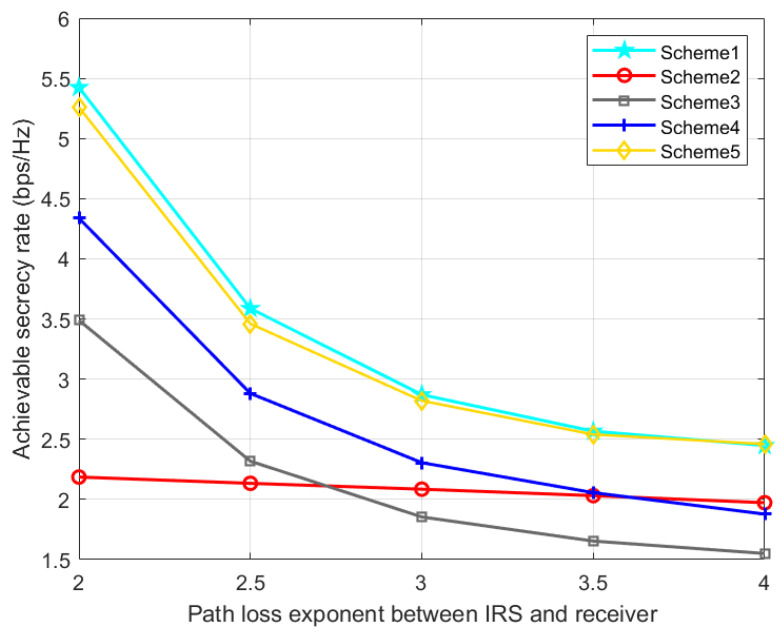
Achievable SR versus the path loss exponent between IRS and receiver.

**Figure 11:**
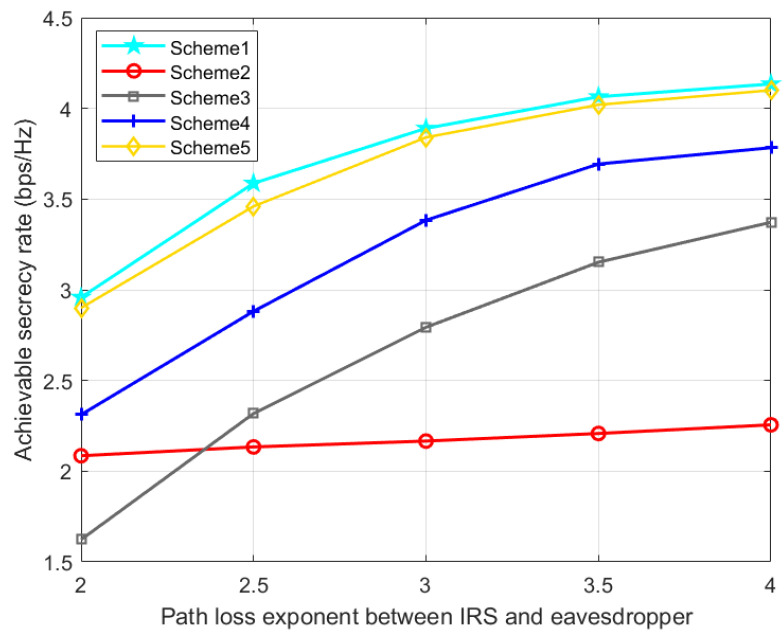
Achievable SR versus the path loss exponent between IRS and eavesdropper.

Figure 10 and Figure 11 show the effect of IRS-Bob and IRS-Eve channel path loss exponents on SR. With the increase of $c_{RB}$, the IRS’s signal reflection strength at Bob gradually decreases, resulting in a decrease in the SR. With the increase of $c_{RE}$, Eve will receive fewer signals from the IRS, which increases the SR. Therefore, when the quality of the reflected legal channel is good, the IRS can promote the secrecy performance of the communication system, but if the quality of the reflected eavesdropping channel is better than that of the reflected legal channel, the deployment of the IRS may be counterproductive. Our proposed scheme 1 is slightly better than scheme 5, and consistently perform better than the other three benchmark schemes.

## 5. Conclusions

In this paper, we investigated the secure communication of an IRS-assisted UAV-MIMOME wireless system. The TPC matrix, AN matrix, IRS phase shift matrix, and UAV position were jointly designed to maximize the achievable SR under the transmit power, the IRS phase shift unit modulus, and the maximum UAV moving distance constraints. Since the problem was non-convex, we proposed an AO algorithm to optimize these variables alternately. The optimal TPC matrix and AN matrix were solved by the Lagrange dual method. The optimal IRS phase shift matrix was solved by three algorithms of ADMM, MM, and RCG. An SRG method was proposed to iteratively optimize the UAV position. Finally, simulations proved the effectiveness of our proposed algorithm and the important role of IRS in UAV-MIMO secure communication. Additionally, our work can be extended to more general multicast scenarios. In our future work, we will investigate the robust and secure design of IRS-assisted UAV MIMO networks considering imperfect eavesdropping CSI.

## Data Availability

Not applicable.
